# Ultrasound modalities and age-specific normative findings in pediatric and adolescent gynecology

**DOI:** 10.3389/frph.2026.1953961

**Published:** 2026-10-06

**Authors:** Martina Asia Policriti, Nina Cooper, May-ai Seah, Bara’a Elhag, Dirk Timmerman, Tom Bourne, Maya Al-Memar

**Affiliations:** 1 Queen Charlotte’s and Chelsea Hospital, Imperial College Healthcare NHS Trust, London, United Kingdom; 2 Department of Woman and Child Health and Public Health, Fondazione Policlinico Universitario Agostino Gemelli IRCCS, Università Cattolica del Sacro Cuore, Rome, Italy; 3 Department of Metabolism, Digestion and Reproduction, Imperial College London, London, United Kingdom; 4 Department of Radiology, St Mary’s Hospital, Imperial College Healthcare NHS Trust, London, United Kingdom; 5 Department of Development and Regeneration, KU Leuven, Leuven, Belgium

**Keywords:** adolescent gynecology, normative values, ovary, pediatric gynecology, pelvic ultrasound, pubertal staging, puberty, uterus

## Abstract

Ultrasound is the first-line modality for imaging the female gynecological organs in children and adolescents. Its interpretation relies on understanding the physiological changes in organ appearance from birth through puberty. This narrative review considers different ultrasound approaches including transabdominal, transvaginal, transrectal, transperineal, Doppler and three-dimensional ultrasound, and when magnetic resonance imaging may add useful information. It then describes how normal uterine, ovarian, and endometrial appearances change across development. Although the evidence is drawn mainly from cross-sectional studies using different equipment, measurement methods, and reporting approaches, the studies describe a broadly consistent developmental pattern. A central practical message emerges: thresholds and diagnostic criteria derived from adults translate poorly to the developing pelvis, so interpretation rests not on memorizing cut-offs but on knowing what to expect at each age and stage, and on recognizing how wide normal physiological variation is. The review also identifies the principal evidence gaps: sparse data at the youngest ages, limited representation of diverse populations, an absence of longitudinal studies, a reliance on adult-derived criteria for diagnosing pathology such as adnexal masses, adenomyosis and endometriosis, and the lack of a standardized reporting framework. It then sets out priorities for future research.

## 1 Introduction

Ultrasound is the first-line investigation of the pediatric and adolescent female pelvis (1–4). It is non-ionizing, widely available, relatively inexpensive, and can usually be performed without sedation (1, 5). Real-time imaging permits dynamic assessment of the uterus, ovaries, adnexa, and adjacent pelvic structures, and the examination is generally well tolerated when performed using a developmentally appropriate technique (6, 7). Transabdominal ultrasound is usually the first approach and is applicable at every age from the neonatal period through adolescence, with the other ultrasound techniques used for defined indications (1–4, 6). Magnetic resonance imaging (MRI) provides valuable complementary information where anatomy is complex or ultrasound findings are equivocal (2, 5).

Interpretation of pelvic ultrasound findings can be made easier by understanding normal developmental anatomy. The female internal genitalia undergo substantial morphological change from birth to reproductive maturity, influenced by a shifting hormonal milieu: transient activation of the hypothalamic–pituitary–gonadal axis during the neonatal period (8), a relatively quiescent prepubertal phase, and progressive reactivation of the gonadal axis during puberty (Figure 1) (3, 4, 6). Uterine size and configuration, the relationship between the uterine corpus and cervix, ovarian volume and follicular pattern, and endometrial visibility all vary with age and pubertal stage (2, 9). Knowing the expected appearances at each stage helps clinicians recognize pathology without overcalling normal development and allows them to reassure patients and families when findings are physiological (1, 6, 10, 11). Physiological variation is wide at every age, so in practice, interpretation is largely a matter of matching what is seen to the child in front of you against her stage of development.

**Figure 1:**
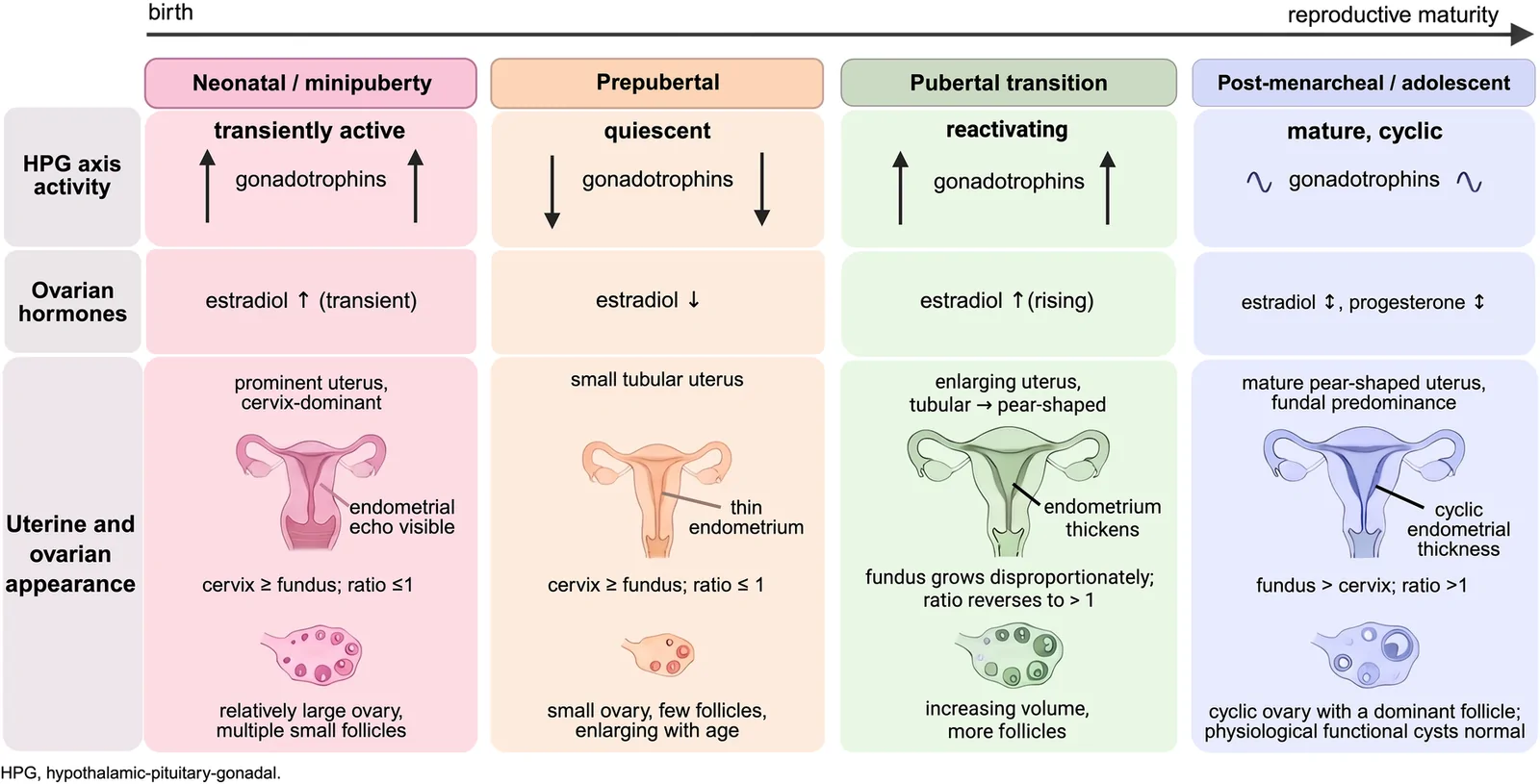
Hormonal influences on uterine and ovarian appearance across developmental stages. Created in BioRender. Policriti, M. (2026) https://BioRender.com/yw2cfnf.

Normative sonographic data for the pediatric female pelvis have accumulated over several decades, but the evidence base is heterogeneous and unevenly distributed across age groups, populations, and imaging methods (12–14); the range of available ultrasound approaches (transvaginal, transrectal, transperineal, three-dimensional, and Doppler) has likewise expanded faster than guidance on their roles in children (4, 7). This review brings together in one place the ultrasound routes available in this population, the normative findings expected at each age and pubertal stage, and the practical considerations that determine whether an examination is diagnostic. It is organized by modality and by developmental stage rather than by presenting complaint, and reported values are set against the measurement conventions that produced them. The practical message throughout is that adult thresholds often do not fit the developing pelvis.

### 1.1 Literature search strategy

PubMed was searched from June to July 2026. No publication date limits were applied; included articles span from 1983 to 2026. Filters for English language and humans were applied. Searches combined MeSH and free-text terms. Eight thematic searches were run, covering review articles on pediatric and adolescent gynecologic ultrasound; normative and reference values; pubertal and developmental change; specific ultrasound modalities; scanning technique and patient preparation; Müllerian, urogenital and associated renal anomalies; physiologic and functional ovarian cysts and related ovarian variants; and cervical and general normal variants and interpretive pitfalls. Both American and British spellings were included where relevant. Full search strings are provided in Supplementary Table 1.

Articles were eligible if they addressed ultrasound assessment of the pediatric or adolescent pelvis, including age- or pubertal-stage-specific uterine, cervical, endometrial or ovarian findings; normative measurements; comparison or description of ultrasound modalities; or practical interpretation, normal variants and pitfalls. Priority was given to healthy-population normative studies, landmark papers, validated reference models, guidelines and consensus documents. Pathology-specific studies and special populations were included only where they informed ultrasound interpretation, modality selection, differential diagnosis or evidence gaps. Pregnancy, obstetric and infertility literature was excluded at the search stage in the review, normative and pubertal searches. Adult-only studies, non-ultrasound imaging studies with no bearing on ultrasound interpretation, and conference abstracts lacking sufficient methodological detail were excluded at screening.

Reference lists of key articles and reviews were screened by hand, and issuing-body websites were searched for relevant guidance and standards. Several clinical themes, including examination anxiety, ovarian torsion, adnexal-mass stratification and disorders of sex development, were identified through this route rather than through the primary searches. Selection and synthesis were guided by relevance to the review aims and authors’ judgment rather than by a predefined algorithm. As this was a narrative review guided by SANRA principles, no protocol was registered, and no PRISMA flow diagram, meta-analysis, or formal risk-of-bias assessment was undertaken. Evidence was synthesized narratively by ultrasound modality, developmental stage, age-specific normative findings, practical interpretation, and gaps in the literature.

## 2 Ultrasound modalities in pediatric and adolescent gynecology

Several ultrasound approaches are available, and each has a practical role in the assessment of the pediatric and adolescent female pelvis. Guidance on choosing between them is currently spread across the literature (5, 7). Table 1 summarizes the available modalities with their principal indications, advantages, limitations, and consent considerations.

**Table 1:** Ultrasound modalities in pediatric and adolescent gynecology: indications, advantages, limitations, and consent considerations.

Modality	Indications	Advantages	Limitations	Consent/comfort
**Transabdominal (TAUS)**	First-line at every age for all indications; initial modality in the emergency setting (acute pain, suspected torsion).	Non-ionizing, widely available, sedation not routinely needed; real-time; applicable across all ages using the filled bladder as an acoustic window.	Operator-dependent; degraded by bowel gas, obesity and over-distension. Non-visualization of an ovary is common in young children (neither seen in ∼35% of neonates, ∼3.5% post-menarche) and does not equal absence.	Non-invasive. Age-matched transducer; warm gel and distraction aid tolerance.
**Transvaginal (TVUS)**	Post-menarcheal adolescents who consent, when TAUS is inadequate. Feasibility is informed by tampon use, prior vaginal examination and sexual history.	Highest spatial resolution of the uterus, endometrium, and ovaries; improves ovarian visualization in post-menarcheal patients over TAUS alone.	Not used in children; in adolescents it is selective rather than routine. No study has compared transvaginal and transabdominal diagnostic performance in children.	Assent or consent from the young person, and a chaperone; parental consent as required by age, capacity and local law. TAUS before TVUS, particularly without prior tampon use or intercourse.
**Transrectal (TRUS)**	Adolescents in whom TAUS is non-diagnostic and transvaginal scanning is not appropriate or not agreed to.	Higher resolution than TAUS (reported subjectively); no bladder filling.	Invasive and rarely used; provokes significantly greater anxiety than TAUS, driven by anticipated rather than experienced pain.	Assent or consent from the young person, and a chaperone; parental consent as required by age, capacity and local law. Attempt TAUS first.
**Transperineal / translabial**	Distal genital tract: imperforate hymen, vaginal atresia, hydro/mucocolpos in neonates and hematocolpos after menarche, transverse vaginal septum, urogenital sinus, perineal mass, DSD; suspected vaginal foreign body; adjunct when TAUS is insufficient.	Reaches the distal vagina and perineum; painless, no bladder preparation, feasible in newborns.	Limited to the distal pelvis; does not show the fundus or ovaries; underutilized.	External approach; consent and chaperone as for a perineal examination.
**Doppler (uterine artery)**	No established indication in routine practice. Investigated as an adjunct marker of pubertal onset. Color Doppler is used separately within a gray-scale examination for adnexal vascularity, parauterine vessels, torsion, and suspected adenomyosis (Sections 2.5, 4.4, 4.5).	Adds hemodynamic information to gray-scale assessment; the uterine artery pulsatility index falls with pubertal onset.	Operator-dependent; no validated cut-off for pubertal onset; published values range widely (2.5–4.6) and come from referred rather than general-population samples. The index falls at puberty but rises again afterwards, so one reading cannot place a girl on the pubertal curve. Measurement fails in about one in five girls, mostly the youngest, and uterine and ovarian volume discriminate pubertal onset better.	Standard add-on to a transabdominal examination; no additional consent.
**Three-dimensional (3D), transabdominal**	Uterine morphology and Müllerian-anomaly characterization (coronal reconstruction); volumetric uterine and ovarian assessment.	Adds the coronal plane; may approach MRI and reduce the need for it; same transabdominal acquisition as 2D.	Operator-dependent and motion-sensitive; needs bladder filling; US volumes (2D and 3D) read systematically higher than MRI; the two are not interchangeable; underutilized. The European 3D reference standard is transvaginal and does not transfer to children or to adolescents in whom transvaginal scanning is not appropriate.	Non-invasive; transabdominal probe, as for 2D.

TAUS, transabdominal ultrasound; TVUS, transvaginal ultrasound; TRUS, transrectal ultrasound; MRI, magnetic resonance imaging; DSD, disorders of sex development. Sources: Transabdominal (1, 2, 6, 15); Transvaginal (1, 2, 22, 23); Transrectal (27, 28); Transperineal/translabial (7, 21, 29, 30); Doppler (31, 32, 33, 34, 35, 65); Three-dimensional (19, 20, 37, 40).

### 2.1 Transabdominal ultrasound

Transabdominal ultrasound (TAUS) is the first-line modality for imaging the pediatric and adolescent female pelvis (1–4, 6) and gives a wide field of view of the uterus, ovaries, and adjacent pelvic structures (2, 5).

Transducer selection should be adapted to the patient’s age and body habitus: high-frequency linear or convex probes (4–12 MHz) in newborns and young children, convex 5–7.5 MHz probes in older girls, and convex 3.5–5 MHz probes in adolescents, in whom greater tissue depth requires lower frequencies for adequate penetration (Figure 2) (6, 7, 15).

**Figure 2:**
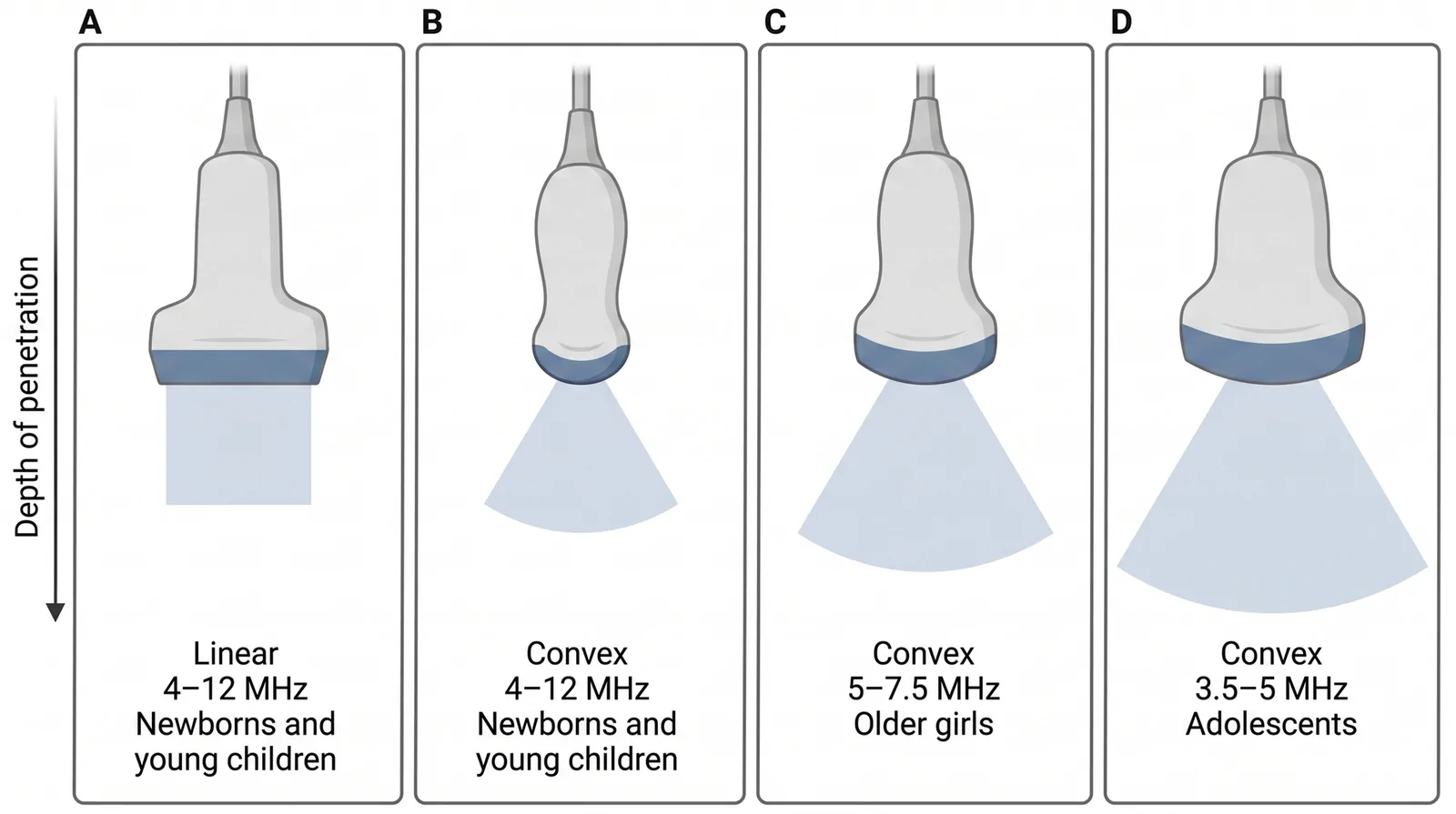
Ultrasound transducer selection in pediatric and adolescent pelvic imaging. **(A)** Linear and **(B)** convex high-frequency transducers (4–12 MHz) are used in newborns and young children; **(C)** a convex transducer of 5–7.5 MHz in older girls; and **(D)** a convex transducer of 3.5–5 MHz in adolescents, in whom greater tissue depth requires lower frequencies for adequate penetration. Beam shape and depth are shown schematically: the linear array produces a rectangular field and the curved arrays a sector field (6, 7, 15). Created in BioRender. Policriti, M. (2026) https://BioRender.com/5trwmyh.

Despite its central role, TAUS has recognized limitations. Image quality may be compromised by bowel gas interposition and obesity (2, 6), as may bladder filling at either extreme: underfilling is a common cause of suboptimal examinations, particularly in prepubertal girls (6), while overdistension compresses the uterus and masks the normal fundal prominence until partial voiding (3, 6, 15). Evidence-based tools for optimizing bladder preparation are discussed in Section 4 (16). Ovarian non-visualization is common, with implications for the exclusion of adnexal torsion discussed in Section 4 (1, 17). Small endometrial or myometrial abnormalities and detailed uterine morphology are better assessed transvaginally (18) or in three dimensions (19, 20). Most data on diagnosis and characterization of pathology such as adenomyosis, endometriosis and adnexal lesions are based on TVUS, with little on the reliability of TAUS in diagnosing gynecological pathology (21, 22).

### 2.2 Transvaginal ultrasound

Transvaginal ultrasound (TVUS) provides the highest spatial resolution of the uterus, endometrium, and ovaries and is the standard approach in adult gynecological imaging (2, 18). In adolescents, however, TVUS is used selectively rather than routinely, when clinically indicated and acceptable to the patient, usually after TAUS. Previous sexual intercourse, regular tampon use, or prior tolerance of vaginal examination may inform feasibility; the decision should be individualized and based on explicit assent or consent (2, 22, 23). The American College of Obstetricians and Gynecologists recommends that transabdominal pelvic ultrasonography should be performed before proceeding with transvaginal ultrasonography in adolescents, particularly for those who have not used tampons or had sexual intercourse (23). When transvaginal ultrasound is nonetheless considered in this group, the young person should understand what the procedure involves, its alternatives, and its benefits and risks, and a chaperone should be offered, with parental consent as required by age, capacity and local law (21, 24–26). Adolescents may feel unable to decline an invasive examination and should be told explicitly that they can refuse or stop at any point (21); consent is therefore best discussed without time pressure, ideally before the patient enters the scanning room (25).

In an early series from a children's hospital, Bellah and Rosenberg reported that TVUS provided better image quality than TAUS in 85% of cases and yielded additional useful information in 58% of examinations (40 examinations in 35 girls) (18). It is important to note, however, that this study was published over three decades ago and reflects the imaging technology and clinical practice of that era; contemporary data on TVUS in the adolescent population are sparse.

### 2.3 Transrectal ultrasound

When TAUS is non-diagnostic or inconclusive and the clinical question requires higher-resolution imaging of the pelvic organs, transrectal ultrasound (TRUS) may be considered as an alternative in adolescents for whom transvaginal ultrasound is not appropriate or not agreed to (27). Güdücü et al. reported that TRUS provided superior image quality compared with TAUS in this setting (27). However, this conclusion was based on a single-center retrospective series, and the comparison was subjective rather than formally quantified.

Because TRUS involves internal probe insertion, particular attention should be paid to comfort and anxiety. Anxiety is significantly greater than with TAUS and appears to be driven more by anticipated than experienced pain (Section 4) (28). Given these findings, TRUS should be reserved for cases in which transabdominal imaging is insufficient and the clinical question cannot be deferred or addressed by alternative means (27, 28). TVUS can be offered as an alternative to TRUS when TAUS has not been as informative (21, 22). A chaperone should be offered and the young person's own assent or consent obtained; whether parental consent is additionally required depends on her age and capacity and on local law (21, 24, 25).

### 2.4 Transperineal and translabial ultrasound

When assessment of the distal genital tract is required, transperineal (or translabial) ultrasound (TPUS) provides a valuable adjunct to TAUS. Performed by placing a high-frequency transducer on the perineum or between the labia, TPUS offers high-resolution imaging of the vagina, urethra, and perineal structures without requiring bladder filling or internal probe insertion (Figure 3) (29). The technique is painless, non-invasive, and can be performed with a standard high-frequency linear transducer (typically 7–12 MHz), with a lower-frequency convex transducer (5–7 MHz) for deeper structures in older children (29).

**Figure 3:**
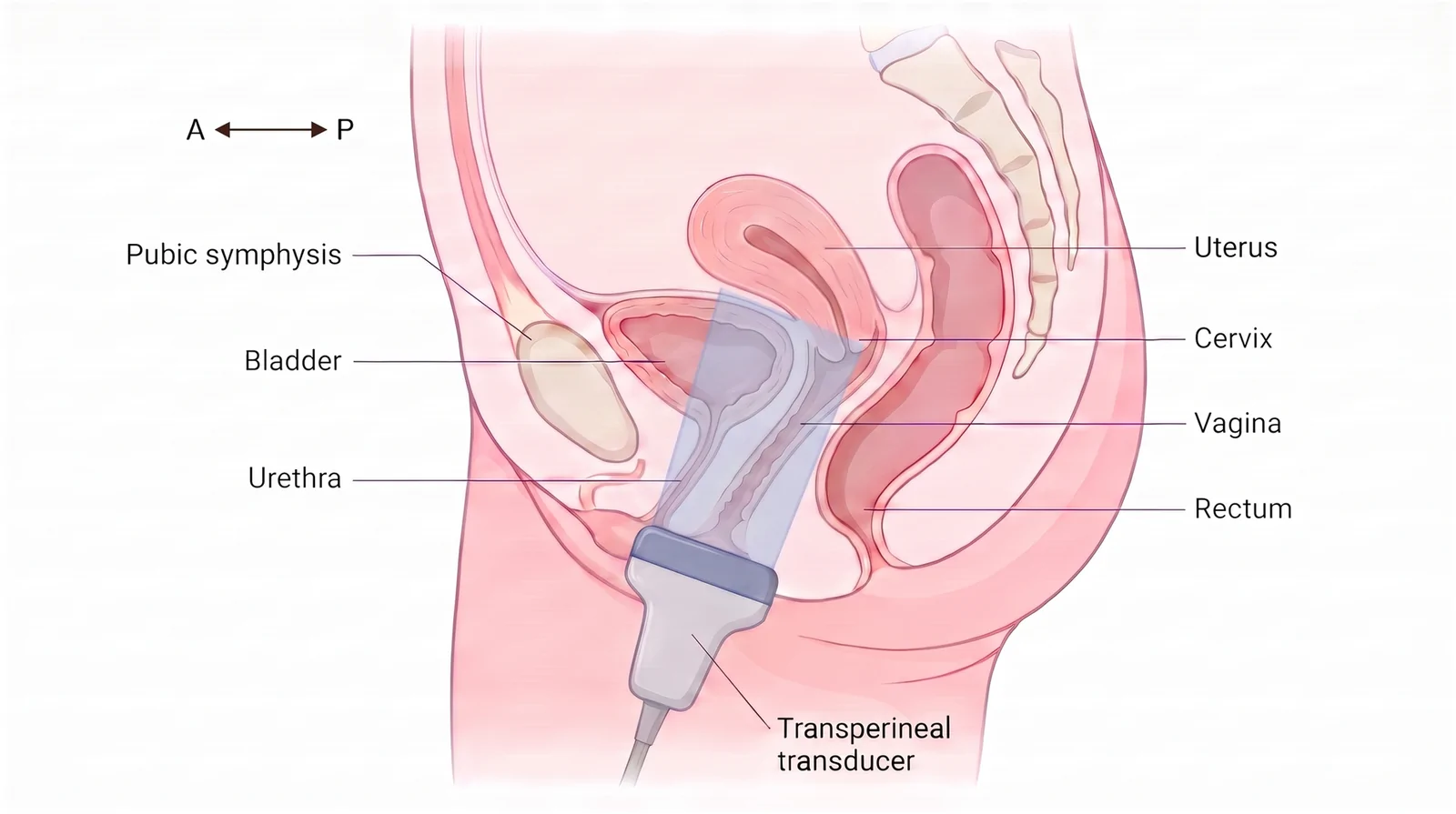
Transperineal ultrasound of the female pelvis. Midsagittal schematic showing a high-frequency transducer placed externally on the perineum. The technique images the vagina, urethra and perineal structures without the need for bladder filling or internal probe insertion (29). Created in BioRender. Policriti, M. (2026) https://BioRender.com/xde30dj.

TPUS is particularly useful in neonates and young children for the evaluation of suspected imperforate hymen, vaginal atresia, hydro- or mucocolpos, urogenital sinus and perineal masses (2, 6, 7, 29). The same approach is used after menarche where an obstructive anomaly presents with hematocolpos, and for measuring a transverse vaginal septum before surgery (29). Combining TAUS and TPUS also improves detection of a vaginal foreign body, for which transabdominal sensitivity alone is 33%, rising to 81% when a transabdominal and transperineal approach is used (21). It also plays an important role in the assessment of disorders of sex development (DSD): the European Society of Pediatric Radiology task force has recommended that pelvic ultrasound in neonates with suspected DSD should include both transabdominal and transperineal approaches, using adapted high-resolution transducers to systematically evaluate the pelvic cavity, genital folds, and inguinal regions (30). Although useful, TPUS is still underutilized and unfamiliar to many practitioners (29), and no pediatric training standard or learning-curve study has yet been published; what learning-curve evidence exists comes from adult urogynecological pelvic-floor imaging, in a context that does not transfer to pediatric genital anatomy. Its field of view is limited to the distal pelvis, and it does not replace TAUS for the assessment of the uterine body, fundus, or ovaries.

### 2.5 Doppler ultrasound

Doppler ultrasound of the uterine arteries provides functional hemodynamic information that complements gray-scale morphological assessment. Uterine artery flow velocity increases through pubertal development, reflecting increased blood flow to the rapidly growing uterus (31). The pulsatility index falls with the onset of puberty (32), rises again once puberty is complete (31), and reported values vary widely across studies (Section 3) (33).

It has been explored as an adjunct in the evaluation of pubertal disorders (32, 34), but no cut-off has been validated, measurement fails in approximately one in five girls (disproportionately the youngest) (31), and no unifying international guidance exists on its use (35). Uterine artery Doppler therefore has no established role in routine clinical practice, and is discussed here for the physiological and normative trajectory it illustrates rather than as a clinically indicated measurement.

That limitation concerns uterine artery Doppler in the assessment of normal pubertal development, not Doppler in general. Color and power Doppler are used when pathology is suspected, and are referred to throughout this review: distinguishing prominent parauterine vessels from follicles (6); assessing vascularity when characterizing an adnexal lesion, where color score is one of the IOTA descriptors applied in pediatric cohorts (1); in suspected torsion, where the limitations are set out in Section 4.5 (15, 21); and in adolescents scanned for suspected adenomyosis or deep endometriosis, where a Doppler-negative intramyometrial cyst is a direct sign of focal adenomyosis and deep lesions are typically avascular (36).

### 2.6 Three-dimensional ultrasound

Three-dimensional ultrasound (3D US) acquires a volumetric dataset that can be reconstructed in any imaging plane, including the coronal view of the uterus, which is often difficult to obtain with conventional two-dimensional scanning (19, 37). This capability is of particular value for the assessment of uterine morphology and the characterization of Müllerian anomalies, where the coronal view can delineate the external fundal contour and the configuration of the uterine cavity with a level of detail that may approach that of magnetic resonance imaging (19). This is particularly relevant in the pediatric and adolescent setting, since some types of Müllerian anomalies characteristically become symptomatic around puberty and menarche, when accurate delineation of uterine and vaginal anatomy is most needed (38). Adding 3D imaging to standard transabdominal ultrasound can significantly increase diagnostic yield and may reduce the need for additional imaging (19), the technique is feasible in infants and children using pediatric transducers (37) and in adolescents, in whom it has been applied to a cohort of 317 post-menarcheal girls (39).

In the research setting, 3D transabdominal US has been used to obtain volumetric measurements of the uterus and ovaries in healthy girls. Hagen et al. found close agreement between 3D and conventional ellipsoid ultrasound volumes (r = 0.931); ultrasound correlated strongly with MRI for uterine volume (r = 0.891) but read systematically higher, so the two are not interchangeable, and agreement for endometrial thickness was weaker (40). Its value lies in coronal morphological assessment and the characterization of Müllerian anomalies (19) rather than in volumetric accuracy, for which it does not outperform two-dimensional transabdominal measurement (Section 5.4). The transvaginal route underlies the 3D technique validated as the reference standard for uterine anomalies in adults, with the transabdominal or transrectal route sanctioned by consensus as the alternative (20). Evidence is currently insufficient to determine whether transabdominal 3D ultrasound provides diagnostic accuracy comparable to the transvaginal reference standard for Müllerian anomaly detection in children and adolescents. Despite this evidence, 3D US remains underutilized in routine pediatric clinical practice (19). Current limitations include dependence on operator expertise, susceptibility to motion artifact in younger children, and the need for adequate bladder filling when performed transabdominally (19).

### 2.7 Modality selection: a stepwise, patient-centered approach

Modality selection should be stepwise and patient-centered, guided by the clinical question, the patient's age and developmental stage, body habitus, and, critically, the comfort and wellbeing of the child (1–3). TAUS is first-line for most indications, with TVUS, TRUS, TPUS and 3D ultrasound reserved for the scenarios set out in Table 1, and MRI a valuable adjunct when findings are equivocal or complex Müllerian or obstructive anomalies are suspected (Figure 4) (19, 23). Diagnostic yield depends as much on operator expertise, patient preparation, and a developmentally sensitive approach as on the modality itself (5, 6).

**Figure 4:**
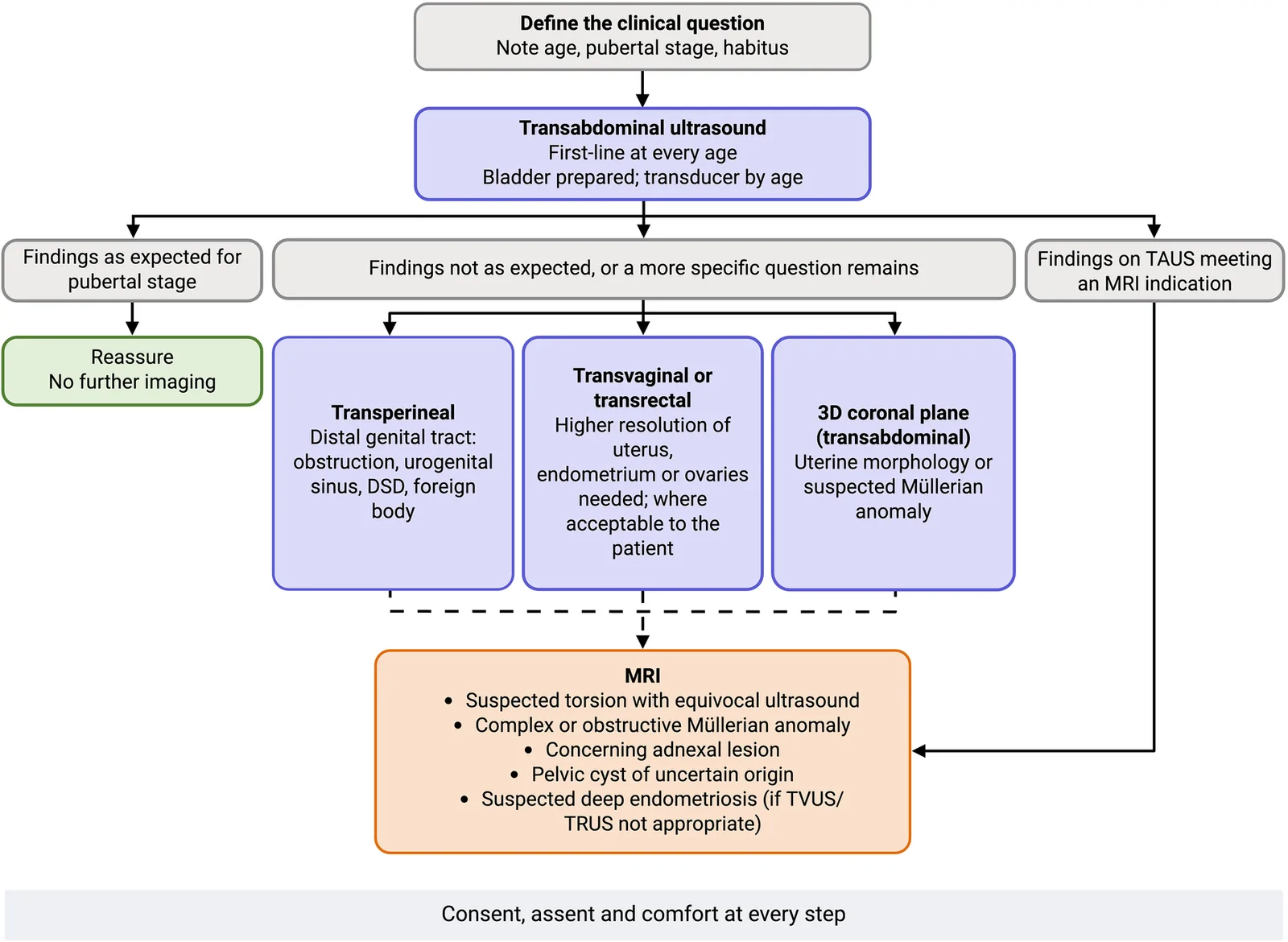
Stepwise selection of ultrasound modality in pediatric and adolescent gynecology. The clinical question, together with the patient's age, pubertal stage and body habitus, guides the examination. Transabdominal ultrasound is first-line at every age, with attention to bladder preparation and a transducer matched to age and habitus. Its findings determine what follows. Where they are as expected for pubertal stage, no further imaging is required. Where they are not as expected, or a more specific question remains, one or more additional ultrasound routes are added at the same examination, chosen by the clinical question: transperineal for the distal genital tract, transvaginal or transrectal where higher resolution of the uterus, endometrium or ovaries is needed and the approach is appropriate and acceptable to the patient, and three-dimensional coronal reconstruction for uterine morphology or a suspected Müllerian anomaly. Three-dimensional imaging is shown transabdominally because the transvaginal route that underlies the validated reference standard for uterine anomalies is often not applicable in this population. MRI may follow any of these routes, or be reached directly from the transabdominal examination where the findings themselves indicate it. Its indications are set out in Section 4.5, and the dashed connector indicates that MRI follows only where one of those indications is met. Indications, advantages, limitations and consent considerations for each modality are given in Table 1. DSD, disorders of sex development; MRI, magnetic resonance imaging; TAUS, transabdominal ultrasound; TRUS, transrectal ultrasound; TVUS, transvaginal ultrasound. Created in BioRender. Policriti, M. (2026) https://BioRender.com/9asn7w6.

## 3 Normal development and expected ultrasound appearances by stage

The ultrasound appearances of the uterus and ovaries change in a predictable sequence from birth to reproductive maturity, reflecting the hormonal changes at each stage (Figure 1). Chronological age is a useful starting point, but pubertal stage often provides better developmental context because girls of the same age may be at different stages of puberty and therefore have different expected pelvic morphology (41). The expected ultrasound appearance should therefore be judged using age, pubertal stage, and hormonal context together. This approach helps distinguish normal variation from appearances altered by abnormal hormonal stimulation or suppression (2, 42). Recognizing this wide but predictable spectrum of normal at every age is what makes physiological interpretation, for the most part, straightforward.

Normative ultrasound measurements of the uterus and ovaries in healthy girls have been reported from birth through adolescence. However, direct comparison across studies is complicated by differences in study design, population, imaging technology, and measurement technique, and these limitations are considered in detail in Section 5. In a meta-analysis of thirteen studies, probe frequency was the principal source of between-study heterogeneity, followed by publication year (43). Despite this heterogeneity, the available evidence describes a coherent developmental trajectory that can be synthesized into age- and stage-specific expectations for clinical practice. Typical appearances and interpretive pitfalls by stage are summarized in Table 2, and published reference ranges in Table 3. Representative sonographic appearances at each stage are shown in Figures 5, 6.

**Table 2:** Age-specific and Tanner stage-specific normative pelvic ultrasound findings from neonates to adolescents.

Stage	Uterus and corpus–cervix	Endometrium	Ovary and follicles	Key interpretation pitfall
**Neonate** *0*–*∼1 month*	Transiently enlarged; cervix ≥ fundus (fundal-to-cervical ratio ∼1 or below).	Thin echogenic stripe often visible.	Relatively large; multiple small follicles; neither ovary seen in ∼35% on TAUS.	Do not mistake the prominent uterus, visible stripe or small follicles for pathology; a non-visualized ovary is not absent.
**Infants and toddlers** *∼1 month–2 years*	Involutes to a small tubular organ; cervix ≥ corpus.	Involuted, thin, often not discretely visible.	Transient minipubertal rise peaking at ∼2–4 months, then decline; small follicles persist.	The prominent early-infant ovary and its follicles are physiological; regression is expected.
**Prepubertal** *∼2 years to puberty · Tanner B1*	Tubular; cervix ≈ corpus; grows gradually with somatic growth.	Thin midline echo visible with modern probes but often not measurable.	Volume rises steadily with age through childhood, not static; small follicles normal.	Reported values vary by population; ovarian volume rises with age rather than remaining fixed.
**Peripubertal** *Tanner B2–B4*	Disproportionate fundal growth reverses the ratio; tubular → pear, steepest between B3 and B4.	Thickens progressively; measurable from B2–B3.	Enlarges through puberty; follicle size and number increase with stage.	Dimensions overlap between adjacent stages and no single parameter reliably separates them; uterine length and volume discriminate better than the fundal-to-cervical ratio.
**Post-menarcheal** *Tanner B5*	Mature pear shape with clear fundal predominance.	Cyclical: varies with cycle phase; interpret against the last menstrual period.	Adult-type; varying with cycle phase; multifollicular pattern common and transient; functional cysts common.	Do not diagnose PMOS on morphology alone; ultrasound is not recommended for PMOS diagnosis until eight years after menarche.

TAUS, transabdominal ultrasound; PMOS, polyendocrine metabolic ovarian syndrome (formerly polycystic ovary syndrome). This table summarizes typical normal appearances and interpretive pitfalls by stage; numeric reference values are in Table 3. Appearances overlap between adjacent stages. Sources: Neonate (1, 2, 46, 48); Infants and toddlers (3, 8, 45, 49, 50); Prepubertal (14, 32, 55, 58); Peripubertal (9, 11, 41, 43); Post-menarcheal (12, 39, 68, 70).

**Table 3:** Published reference ranges for uterine, endometrial, and ovarian measurements by age group and pubertal stage.

Stage	Uterine length (cm)	Uterine volume (mL)	Fundal-to-cervical ratio	Endometrium (mm)	Ovarian volume (mL)	Largest follicle (mm)
**Neonate** *0–∼1 mo · Tanner B1*	3.4–4.2 (means)	NR	≤1 (AP diameters)	thin, visible; not measured	0.2 (median, birth)	typically <9
**Infancy** *∼1 mo–2 y*	2.7–2.9 (means)	NR	≤1 (AP diameters)	thin, often not visible	≤1 (medians; peak ∼0.9 at 2–4 mo)	typically <9 (up to 20)
**Prepubertal** *∼2 y to puberty · Tanner B1*	2.5–4 (means)	1.6 (median; range 0.7–7.9)	0.95 (mean; fundus ÷ cervix diameter)	not measurable in ∼88%	0.7–2.5 (means, 2–10 y)	typically <9
**Peripubertal** *Tanner B2–B4*	3.7–6.9 (medians)	2.8–37 (medians)	1.12–1.29 (means; fundus ÷ cervix diameter)	1.0–2.8 (means, B2–B3); cyclical from B4	∼4–8 (means, late puberty)	5.6–8.4 (means)
**Post-menarcheal**	7.4 (median; 10th–90th centile 6.5–8.4)	46.7 (median; 10th–90th centile 32–62)	1.22 (mean; fundus ÷ cervix diameter)	cyclical: week 1 mean 4.5 mm (SD 2.8) to week 4 mean 7.6 mm (SD 3.8)	7.2 (median; 10th–90th centile 4.9–11.3; cycle days 2–5)	NR

These values describe healthy-population variation and are not diagnostic thresholds; individual findings must be interpreted in clinical context. Values come from different studies and are not directly comparable between cells; each numeric entry states whether it is a mean, a median, or a centile interval, and entries without a stated statistic are descriptive. Most are measures of central tendency rather than limits of normal, so the spread within any single study is considerably wider and ranges overlap between adjacent stages, most markedly for the fundal-to-cervical ratio, which ranged from 0.44 to 2.13 across breast stages against means of 0.95 to 1.29, with the ranges at stages 1 and 2 (0.44–1.75 and 0.55–2.00) almost entirely overlapping. Single follicles of 12–21 mm were likewise seen in normal scans against a mean largest follicle of 8.4 mm at B4/5. Figures are values reported in the studies cited in Section 3. Sources by column: Uterine length: neonate (46, 48); infancy-prepubertal (9, 12); B2–B4 (14); post-menarcheal median, 10th–90th centile (39). Uterine volume: median and range (41); prepubertal upper limit generally <4–5 mL (13); a neonatal uterine volume is reported in some series but ranges widely with method [∼1.6 cm^3^ (12); 10.0 cm^3^ (48), the latter from a cylinder rather than ellipsoid model], so no single reference value is given. Fundal-to-cervical ratio: B1–B5 (9), measured as fundus diameter divided by cervix diameter; ∼1 or below in neonates from anteroposterior diameters (46), and below 1 through infancy and early childhood (47). The ratio is measured differently across studies: anteroposterior diameters (46, 47, 56, 60–64), fundus-to-cervix diameter (9), uterine body width to cervix width (4), or a categorical shape judgement rather than a number (55). Values are therefore not interchangeable. At Tanner B2, published means range from 1.1 to 1.36 (9, 61–64). Studies using the same anteroposterior convention span most of this range, so the variation reflects differences between populations and equipment as well as measurement method. Endometrium: B2–B3 means (9), with prepubertal visibility (55, 32, 14); after menarche it is cyclical—cycle-week means rise from 4.5 mm in week 1 to 7.6 mm in week 4, with standard deviations of 2.8–3.8 mm, so individual normal values extend well above the means (12)—and must be interpreted against the last menstrual period. Ovarian volume: model by age (58); neonatal and infant values, birth ∼0.2 mL, peak ∼0.9 mL at 2–4 months (49), corroborated at birth, 0.2 cm³ (48); values to two years (45, 50); post-menarcheal median, 10th–90th centile, scanned on cycle days 2–5, so follicular-phase values (39); pooled adult mean 6.1 mL (66). Largest follicle: typically <9 mm before puberty (4, 6), though larger follicles >9 mm are seen in 18% of follicle-containing ovaries in the first two years (50) and the largest follicle reached 19–20 mm at 4–8 weeks in one infant cohort (49). Peripubertal values are means from two series, one to B4/5 (10) and one premenarcheal to B4 (63). After menarche the largest follicle tracks cycle phase rather than age or stage; reported values span 3–36 mm (41), so no single reference value is given. NR, no standard reference value. Volumes are otherwise ellipsoid-derived; they agree closely with 3D ultrasound but read higher than MRI; values also vary with transducer frequency, era and population. B1–B5, Tanner breast stages; 3D, three-dimensional; MRI, magnetic resonance imaging.

**Figure 5:**
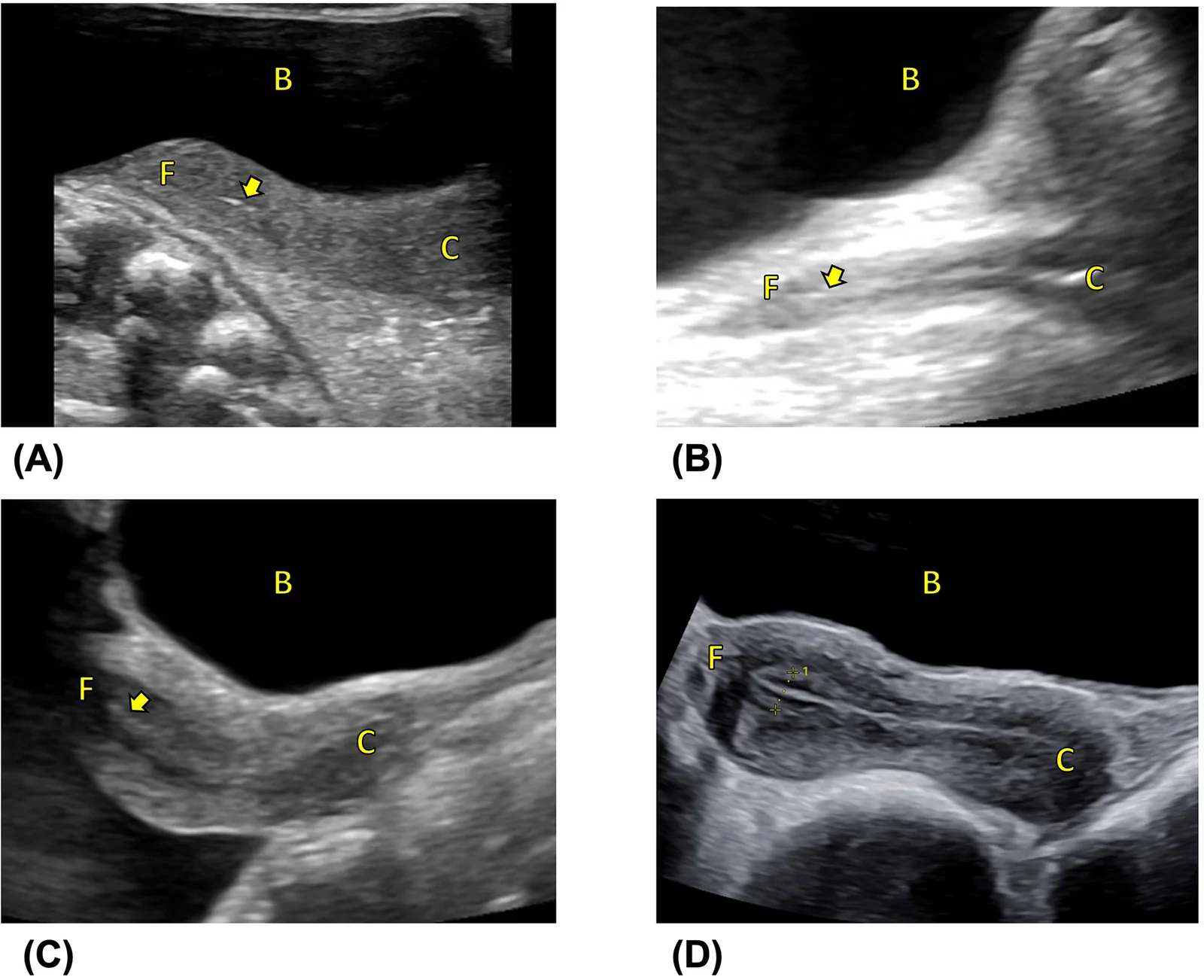
Representative sonographic appearances of the uterus across developmental stages. In each panel the fundus **(F)**, cervix **(C)** and bladder **(B)** are labeled, and the arrow indicates the endometrium. **(A)** Neonatal uterus, with a visible endometrial echo and cervix equal to or greater than the fundus. **(B)** Prepubertal uterus, small and tubular, with a thin midline echo. **(C)** Peripubertal uterus, enlarging with reversal of the fundal-to-cervical ratio and progressive endometrial thickening. **(D)** Post-menarcheal uterus, mature pear-shaped with fundal predominance; calipers indicate endometrial thickness.

**Figure 6:**
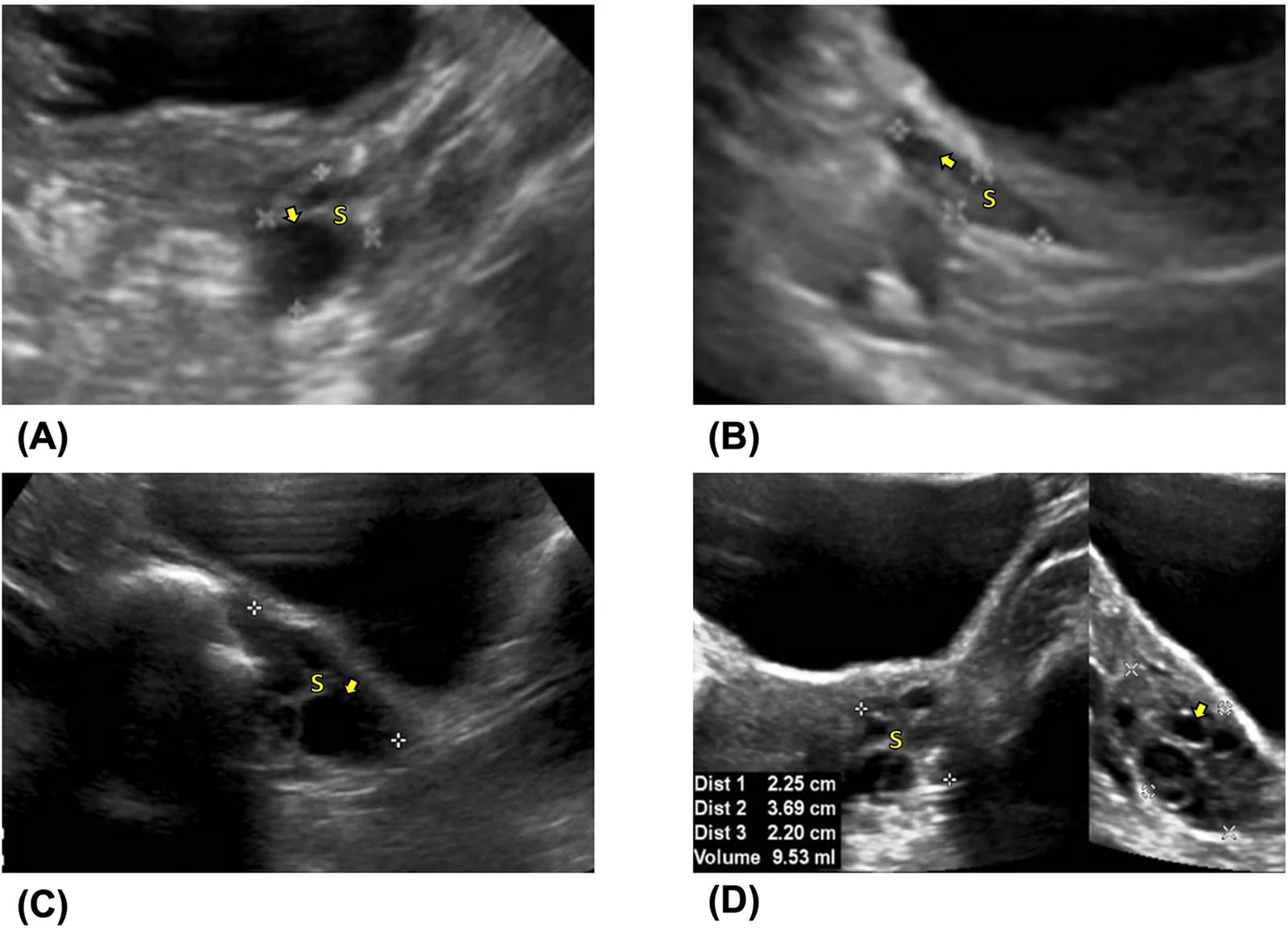
Representative sonographic appearances of the ovary across developmental stages. In each panel the arrow indicates a follicle, S the ovarian stroma, and calipers mark the ovarian margins. **(A)** Neonatal ovary, relatively large with small follicles. **(B)** Prepubertal ovary, small and quiescent, with a small follicle visible. **(C)** Peripubertal ovary, increasing volume with more follicles. **(D)** Post-menarcheal ovary, shown in two orthogonal planes with the calculated volume displayed.

Fundal-to-cervical ratios should be interpreted according to the measurement definition used in each study: some calculate the ratio from anteroposterior diameters, others from the fundus-to-cervix diameter or from uterine body and cervix widths, and some report it as a categorical shape judgement rather than a number.

### 3.1 Neonatal period and minipuberty

The fetal uterus grows steadily through gestation under the influence of estrogens produced mainly by the placenta (8). The fetal hypothalamic–pituitary–gonadal (HPG) axis is itself active in the mid-gestational fetus and is then silenced towards term, attributed to negative feedback from placental hormones, particularly estrogens (44). Withdrawal of maternal hormones at birth removes this restraint, and the axis becomes transiently active over the first months of life, a period termed minipuberty (8, 44). As minipuberty resolves and reproductive hormones decline, the uterus involutes and ovarian follicular activity subsides toward its low prepubertal baseline (2, 8, 45).

The neonatal uterus is transiently stimulated by maternal and placental estrogens and is enlarged relative to later infancy, with a frequently visible endometrial echo (2, 46). A small amount of fluid within the endometrial cavity is seen in roughly a quarter of neonates and is physiological, reflecting maternal-hormone withdrawal; it should be distinguished from an obstructive collection such as hydrometrocolpos (4, 46). In contrast to the fundal dominance that develops at puberty, the neonatal cervix is equal to or greater than the fundus, giving a fundal-to-cervical ratio of approximately 1 or below (2, 46, 47). Mean uterine length in the first days of life has been reported at approximately 3.4–4.2 cm (46, 48), and median ovarian volume rises from ∼0.2 mL at birth to a peak of ∼0.9–1.0 mL between two and four months, before declining (49); one recent cross-sectional cohort reported a lower median of ∼0.3 mL (8). Neonatal ovaries commonly contain multiple follicles, typically less than 9 mm in diameter (50). Fischer et al., in a cross-sectional study of 302 infant girls at minipuberty, found that reproductive hormone concentrations correlated with antral follicle count and that circulating estradiol correlated with endometrial thickness (8), while Chin et al., in a longitudinal cohort followed from birth to nine months, documented a rise in ovarian volume and follicle number to a peak around four months, followed by a decline (49). Visible intrauterine fluid was present in about a third of infants (35%) and minor vaginal bleeding in a small minority (around 6%) during minipuberty, both normal responses to transient estrogen exposure and its withdrawal (8). In practice, these hormonally stimulated uterine and ovarian appearances are usually physiological during minipuberty when they fit the clinical and hormonal context.

Many structural anomalies of the female genital tract originate in fetal life, when the paired Müllerian ducts fuse and canalize and the ovaries form and descend; disturbance of these processes underlies obstructive and complex Müllerian anomalies (51). Some are recognized antenatally, hydro(metro)colpos and fetal ovarian cysts being among the more commonly detected, usually in the third trimester, allowing planned neonatal assessment (52). A difference of sex development may likewise be suspected before birth when the sex predicted by non-invasive prenatal testing does not correlate with the phenotype seen antenatally or at birth, prompting coordinated pelvic and gonadal imaging in the neonatal period (53).

### 3.2 Later infancy and early childhood

By approximately 12–24 months of age, the uterus transitions to a tubular morphology in which the cervix is equal to or slightly larger than the corpus, uterine length falls to a mean of approximately 2.7 cm (12), and the involuted endometrium is thin and often not discretely visible (3). Ovarian volume similarly decreases, generally around or below 1 mL through the first year, with variation across studies (45, 50).

Follicles may persist throughout infancy and are a normal finding; Cohen et al. identified follicles in 84% of ovaries examined in infants aged 1 day to 24 months, with most measuring less than 9 mm, though larger follicles are not rare: follicles exceeding 9 mm were present in 18% of the ovaries containing follicles (50), and in a longitudinal infant cohort the largest follicle had a median diameter of 8 mm at four weeks and 7 mm at eight weeks, with occasional infants reaching 19–20 mm (49). Follicles in infancy are therefore part of normal ovarian activity and should not automatically be labeled as pathological stimulation or true ovarian cysts (2, 50).

### 3.3 Prepubertal years

Following the resolution of minipuberty, the HPG axis enters a period of relative quiescence that persists until the onset of puberty. Between approximately two years of age and the onset of puberty, the uterus and ovaries change gradually rather than remaining completely static. The youngest children in this age group remain less well studied than neonates and peripubertal girls (12, 54). The uterus maintains a tubular configuration with a fundal-to-cervical ratio of approximately 1:1, whether assessed from anteroposterior diameters (21), or as a categorical shape judgement (55), with an earlier series describing slight cervical predominance (56, 57). Reported mean uterine lengths range from approximately 2.5 to 4.0 cm across studies, with gradual increases reflecting somatic growth rather than hormonal stimulation (9, 12), and uterine volume is generally reported below 4–5 mL (12, 13, 45). Prepubertal ovarian volume rises steadily with age, from a mean of 0.7 mL at 2 years to 1.2 mL at 6 years, 1.7 mL at 8 years and 2.5 mL at 10 years (58). Kelsey et al. developed validated normative models for both ovarian volume and uterine volume across the lifespan using pooled data from multiple studies, providing smoothed centile curves that account for the gradual prepubertal increase and the accelerated growth at puberty (13, 58). Follicles are commonly observed in prepubertal ovaries and are considered a normal finding (10, 41, 59). With modern equipment a thin midline endometrial echo is visible in essentially all prepubertal girls (55), although it is frequently too thin to measure (32); in one series the endometrium was not measurable in 88% of prepubertal girls (14).

An important consideration is the substantial variation in reported normative values across populations. Studies from Greece, India, Brazil, the United Kingdom, Spain, Argentina, Iran and Turkey have all reported somewhat different reference ranges for prepubertal uterine and ovarian dimensions, likely reflecting differences in population demographics, nutritional status, body composition, and measurement methodology (14, 47, 54, 55, 60–64). Gilligan et al. provided one of the most comprehensive recent datasets, reporting normative values by year of age in a large cohort and highlighting the need for population-specific reference data (12).

### 3.4 Pubertal transition

Reactivation of the HPG axis around puberty drives progressive growth and maturation of the internal genitalia. Uterine and endometrial growth, being estrogen-dependent, track pubertal stage more closely than ovarian growth (40, 57).

The uterus undergoes progressive enlargement with disproportionate fundal growth, resulting in reversal of the fundal-to-cervical ratio and transition from a tubular to a pear-shaped configuration (9, 21). In a landmark study of 166 healthy girls, Holm et al. demonstrated that median uterine volume increased from 1.6 mL at Tanner breast stage B1 to 43 mL at B5, with the most marked increase between stages B3 and B4 (41). The endometrium thickens progressively through puberty and becomes measurable from Tanner stage B2–B3 onward (9, 40).

Ovarian volume continues to increase through puberty, reaching roughly 4–8 mL in late puberty (58). Follicle number rises with pubertal stage (11), and the largest follicle grows: from a mean of about 4.7 mm at Tanner breast stage 1 to 8.4 mm by stages 4–5 in one series (10), and from 5.2 mm at stage 1 to 7.4 mm at stage 4 in a second (63). Multiple studies have identified measurement thresholds suggestive of pubertal onset, but reported values vary widely: across the literature, cut-offs span 1.07 to 4 mL for uterine volume, 22 to 40.9 mm for uterine length, 0.98 to 1.45 for the fundal-to-cervical ratio, and 1.0 to 3.5 mL for ovarian volume (35). In a large series of healthy girls, uterine volume was the single best discriminator (cut-off 3.75 cm^3^, area under the curve 0.897), marginally ahead of uterine length (3.55 cm, 0.837) and the uterine artery pulsatility index (0.838); ovarian volume and endometrial thickness performed less well (32). No parameter, however, reliably distinguished adjacent pubertal stages: this held for every measure in that cohort (32), a Spanish and an Argentinian cohort likewise found the fundal-to-cervical ratio unchanged in 91% of girls at Tanner 2 with no difference in any measurement between Tanner 1 and 2 (14, 55); an Iranian cohort found the ratio unchanged between B1 and B2 even though every other uterine measurement differed significantly across those stages, and identified no cut-off with both high sensitivity and specificity for any variable (63); and earlier work likewise reported considerable overlap in uterine dimensions between stages (9). Smets et al., in a recent European review, concluded that no properly validated cut-off exists for any sonographic parameter, and that uterine and ovarian appearances overlap between prepubertal and pubertal girls (35).

Uterine artery Doppler has been proposed as a marker of pubertal onset, since the pulsatility index falls with puberty (32), and reported cut-offs span 2.5–4.6 (sensitivity 77%–94%, specificity 85%–100%) (33). Two problems limit its use: the index is not monotonic, rising again after puberty to adult values no different from prepubertal ones (31), and every published cut-off comes from girls referred to specialist clinics rather than from general-population samples (33, 35). A more recent cohort found uterine and ovarian volume markedly outperformed it (area under the curve 0.97 vs. 0.76), so Doppler is best regarded as an adjunct to gray-scale morphometry rather than a standalone marker of pubertal onset (35, 65).

### 3.5 Adolescence and post-menarche

Following menarche, cyclic ovarian hormone production is established, and the sonographic appearances of the uterus and ovaries become correspondingly dynamic. Sustained estrogen exposure completes uterine growth (3, 9), while cyclic estrogen and progesterone drive the endometrium through proliferative and secretory phases (3, 12). In the ovary, the recurring sequence of follicular recruitment, dominant follicle selection, and corpus luteum formation underlies the cyclic change in ovarian volume (12).

The uterus assumes its mature pear-shaped configuration with clear fundal predominance. Reported mean uterine lengths in post-menarcheal adolescents range from approximately 6 to 8 cm, with considerable individual variation (12). In a Danish population cohort assessed by three-dimensional ultrasound, median uterine length was 7.4 cm (10th–90th centile 6.5–8.4), median uterine volume 46.7 cm^3^ (32.2–62.3), and median ovarian volume 7.2 cm^3^ (4.9–11.3), with a median of 20 follicles per girl (10th–90th centile 14–25) (39). The endometrium exhibits cyclic variation in thickness corresponding to the phases of the menstrual cycle, and its appearance should be interpreted in the context of the cycle day when known (3, 12).

Ovarian morphology becomes cyclically dynamic, with the volume of the presumably ovulating ovary peaking in the third week of the cycle, although variation across cycle weeks was not formally tested (12); physiological functional cysts, including simple and hemorrhagic corpus luteum cysts, are common and typically resolve spontaneously (2, 39). Mean ovarian volume is typically reported between 4 and 10 mL, though values vary with cycle phase and measurement technique (12). A systematic review and meta-analysis of the healthy ovary pooled a mean ovarian volume of 6.1 mL and a whole-ovary follicle count of around eight per ovary in reproductive-aged women, and found follicle count to depend significantly on transducer frequency, reinforcing measurement technique as a major source of between-study variation (66).

Polycystic ovary syndrome was renamed polyendocrine metabolic ovarian syndrome (PMOS) in 2026, and implementation is proceeding over a three-year transition during which both terms remain in use (67). The 2023 international evidence-based guideline recommends that pelvic ultrasound not be used to diagnose PMOS until eight years after menarche, when the hypothalamic–pituitary–ovarian axis is considered mature (68); in adolescents the diagnosis rests instead on irregular cycles together with clinical or biochemical hyperandrogenism (69). A multifollicular ovarian pattern is common in the early post-menarcheal years: in a Danish population cohort scanned a median of 2.9 years after menarche, 34% of healthy girls had twelve or more follicles of 2–7.9 mm in at least one ovary, meeting the follicle-count threshold in use at the time, yet only 8.5% met Rotterdam criteria for PMOS, which additionally require hyperandrogenism or ovulatory dysfunction (39). The morphological threshold has since been raised, from twelve follicles of 2–9 mm to twenty per ovary, though it is specified for adults and for higher-frequency transducers (68). A comparable proportion, 43%, met a polycystic morphology definition among 302 healthy Italian girls aged 14–18, though counted per ovarian section rather than per ovary, and assessed largely by higher-frequency transvaginal ultrasound rather than the transabdominal approach used in the Danish cohort, so the two figures are not directly comparable (70). The pattern is transient: it became significantly less frequent after the third post-menarcheal year, while normal ovarian morphology became significantly more common beyond five years (70). Ovarian size and follicle numbers rise rapidly through adolescence and peak in early adulthood, so adult definitions of polycystic ovarian morphology are inappropriate at this stage.

## 4 Practical considerations and clinical application

A few practical adjustments help obtain good-quality pelvic ultrasound images in children and adolescents. Because the internal genitalia change throughout development, findings should be matched to age-related physiology (1). Equally important is the ability to apply normative knowledge in clinical context: recognizing reassuring findings, avoiding over-investigation of normal variants, and identifying when further imaging is warranted (14).

### 4.1 Patient preparation and bladder filling

A well-filled but not over-distended bladder is the most important preparation for TAUS: it provides an acoustic window and displaces bowel from the pelvis (4, 6), achieved by avoiding voiding for an hour beforehand and encouraging oral fluids, approximately 470 mL in teenagers (2). Target volumes are not uniform: an age- and weight-based nomogram derived from 1,030 successful scans in girls aged 8–18 years shows median bladder volume rising with age and weight, ranging at 8–12 years from 91 mL at or below the 75th weight centile to 232 mL above the 97th (16). This nomogram derives from a single-center emergency-department cohort in which younger children were underrepresented and interrater reliability was not assessed, and its authors call for further evaluation before it is applied more widely (16). The patient's own report of fullness is unreliable: 15% of scans ordered on that basis failed at the first attempt, against none when fullness was confirmed objectively (71).

### 4.2 Trauma-informed and developmentally appropriate scanning

Pelvic ultrasound involving the genital region is a source of significant anxiety. Among 51 adolescent–caregiver pairs attending for a first pelvic ultrasound, both transabdominal and transrectal scans provoked high-level anxiety in patients and moderate anxiety in caregivers; because caregiver anxiety may itself worsen a child's reaction, anxiety-reducing measures should be directed at caregivers as well as patients (28). A trauma-informed approach includes age-appropriate explanation, the child's assent or consent with parental consent as required, and the offer of a chaperone (24, 25). Simple measures can make the examination more comfortable: warm ultrasound gel, a calm and child-friendly room, a familiar adult, and age-appropriate distraction such as music or a film. Sedation is not routinely required (4, 6). The approach should be adapted to developmental stage: transducer frequency is matched to the child's age and body habitus (Section 2), and younger children or those who find the examination difficult can often be reassured with a familiar adult present and distraction (6). Across all ages, and particularly for the internal approaches, the child or young person's capacity to consent should be assessed, privacy respected, and their right to decline made explicit (24, 25).

### 4.3 Non-visualization of ovaries and operator dependence

Non-visualization of one or both ovaries is common on pediatric transabdominal ultrasound and becomes markedly less frequent with age. Ultrasound is dependent on the experience of the sonographer or radiologist, and bowel gas and obesity reduce image quality (6).

In a UK referral cohort, neither ovary was identified in 35% of neonates, 15.3% of premenarcheal girls and 3.5% of post-menarcheal girls (1). The opposite may hold for torsion. On the reasoning that a torted ovary is enlarged and should be visible without a distended bladder, non-visualization of the ovaries with a non-distended bladder carried a negative predictive value of 0.997 (95% CI 0.984–0.999) in 341 girls aged 1–19 years, sparing a mean 105 minutes' wait (17). But sensitivity was 88.9% on nine torsions (95% CI 51.8–99.7%), the one missed girl went to theatre on clinical suspicion alone, and the authors caution the rule may hold only after menarche (17). In post-menarcheal patients, adding a transvaginal or transrectal approach where appropriate improves ovarian visualization over transabdominal scanning alone (1, 27).

### 4.4 Recognizing normal variants

Several normal appearances can resemble pathology, but they are easier to recognize when age and pubertal stage are considered. Table 4 summarizes the most common examples and their distinguishing features. Diagnosing a uterine or vaginal anomaly before puberty is unreliable, because the prepubertal uterus is small, the endometrium unstimulated, and the vagina undistended (23); MRI does not help, as uterine zonal anatomy is undefined before puberty (72). Rudimentary Müllerian structures are difficult to interpret sonographically and may be particularly misleading before puberty, and Müllerian agenesis is usually identified only on evaluation for primary amenorrhea (73). A shallow fundal indentation (arcuate configuration) is a common normal variant and should not be over-interpreted as a septate or bicornuate anomaly (40, 72, 74). The neonatal period is a partial exception: the uterus remains prominent under maternal hormonal influence and is identified in the large majority of newborns (46), so a duplication anomaly or an obstructive collection may be appreciated then, particularly when a neonate is screened after renal agenesis or a multicystic dysplastic kidney is found (51). Beyond infancy the small, tubular uterus makes anomalies difficult to evaluate, and reassessment towards the end of puberty is advised (51).

**Table 4:** Common normal variants and their distinguishing features from pathology.

Normal variant (stage)	Normal appearance	Mimics	How to tell it apart, and when to review
**Small follicles at all ages** *All ages*	Small follicles within the ovary at any age; the typical prepubertal ovary is heterogeneous, not homogeneous.	Cystic ovarian pathology.	A normal finding at all ages, typically <9 mm; follicles in 84% of infant ovaries. A discrete cyst with features that are not clearly benign warrants further evaluation (see physiological cysts, below).
**Physiological/functional ovarian cysts** *Peripubertal–adolescent*	Simple cysts and hemorrhagic corpus-luteum cysts; common and usually self-limiting.	Neoplasm.	Managed conservatively with interval imaging at 4–8 weeks; most resolve spontaneously, and a lesion that persists with reassuring features may continue to be observed. Features that are not clearly benign (septations, solid components, vascularity, or a cyst that enlarges) instead warrant further evaluation.
**Multifollicular (“polycystic”) morphology** *Adolescent*	Multiple small follicles giving a polycystic appearance; physiological, especially in the first 1–3 years after menarche.	Polyendocrine metabolic ovarian syndrome (PMOS; formerly polycystic ovary syndrome).	Present in 34%–43% of healthy post-menarcheal girls and transient; report as “multifollicular,” not “polycystic.” Ultrasound is not advised for PMOS diagnosis until eight years post-menarche; in adolescents the diagnosis requires both irregular menstrual cycles and clinical or biochemical hyperandrogenism, not morphology alone.
**Thin or visible endometrium in a prepubertal girl** *Prepubertal*	A thin midline echo visible with modern probes but usually not measurable.	Precocious estrogenization.	Visibility itself does not indicate puberty; the endometrium was not measurable in ∼88% of prepubertal girls. Review for a clearly thick or echogenic endometrium with other precocity signs.
**Arcuate uterus** *Peripubertal–adolescent*	A normal convex external fundal contour with a shallow, rounded saddle-shape indentation of the endometrial canal at the fundus.	Septate or bicornuate uterus.	A common normal variant, found in around 3% of healthy girls; the smooth, shallow indentation and convex outer contour distinguish it from a septate (deep, V-shaped) or bicornuate (external cleft) uterus, and it carries no heightened pregnancy risk. The ASRM 2021 classification groups arcuate with the normal uterus.
**Parauterine vessels** *All ages*	Hypoechoic tubular structures adjacent to the ovary that can appear round in cross-section.	Ovarian follicles or a small cyst.	Prominent parauterine vessels may simulate follicles on gray-scale imaging; color Doppler shows flow within vessels, whereas follicles are avascular.

PMOS, polyendocrine metabolic ovarian syndrome (formerly polycystic ovary syndrome). Each “mimic” is the pathology named in the cited source; physiological findings must be interpreted for age and pubertal stage before pathology is inferred. Sources: Small follicles at all ages (2, 50, 59); physiological/functional ovarian cysts (1, 2); multifollicular (1, 21, 39, 68, 69, 70); thin or visible endometrium (14, 32, 35, 55); arcuate uterus (40, 72, 74); parauterine vessels (6).

As discussed in Section 3, follicles are a normal finding at all ages, physiological simple cysts and hemorrhagic corpus luteum cysts are common in post-pubertal girls (2). Prominent parauterine vessels may resemble follicles or a small cyst, but color Doppler confirms their vascular nature (6). The multifollicular ovarian pattern common in early post-menarcheal girls should not be mistaken for polycystic ovarian morphology, and ultrasound should not be used to diagnose PMOS until eight years after menarche (68); reported prevalences and their methodological caveats are discussed in Section 3.5. Hormonal context matters too: combined oral contraception reduces both ovarian volume and endometrial thickness and should be taken into account when appearances are interpreted (39). Finally, a trace of pelvic free fluid is a normal incidental finding and does not itself indicate pathology (6).

### 4.5 Reassuring findings and indications for further imaging

When findings match the expected appearances for a girl's age and pubertal stage (Section 3, Table 3), they can be treated as reassuring, and benign lesions are often managed by observation rather than further imaging. Not all parameters are equally useful: in a meta-analysis of 13 studies and 1,977 girls referred for early breast development, uterine length best discriminated central precocious puberty from premature thelarche (standardized mean difference 1.38, 95% CI 0.99–1.78), ahead of uterine and ovarian volume and cross-sectional area, while the fundal-to-cervical ratio performed worst (0.90, 95% CI 0.07–1.73) (43). Recognizing normal accurately matters in itself: uncertainty drives unnecessary imaging and surgery, and wrongly labeling an adolescent with PMOS risks medicalization and anxiety (1). In post-menarcheal girls, functional adnexal lesions such as unilocular anechoic cysts and cysts with debris suggesting hemorrhage are followed at four to eight weeks; most resolve, persistent but reassuring cysts can be observed, and increasing size, septations, solid components or vascularity prompt further evaluation (1, 2).

In the emergent setting, ultrasound remains the first-line modality for acute presentations including suspected torsion and can be performed at the bedside as point-of-care ultrasound (15). Doppler findings, however, neither establish nor exclude torsion, since a torted ovary may retain flow through its dual ovarian and uterine supply while a normal ovary may show none, so the gray-scale appearance is the more reliable guide, with the whirlpool sign relatively specific (4, 15, 21).

Adnexal torsion remains a surgical diagnosis: no clinical or imaging criteria confirm it preoperatively, and adolescents with clinical suspicion of torsion should undergo emergent diagnostic laparoscopy (75). Because delay allows progression to irreversible ischemia and necrosis, imaging must not postpone urgent surgical review where clinical suspicion is high, and a normal or equivocal scan does not exclude torsion (15, 17, 21).

For obstructive anomalies, transperineal ultrasound sits between transabdominal ultrasound and MRI: it can measure a transverse vaginal septum’s thickness and distance from the vestibule, needed for surgical planning, but cannot resolve Müllerian agenesis, cervical agenesis or complex uterine anomalies, for which MRI may be required (29).

MRI may therefore be indicated in the following situations:

- Suspected torsion where clinical suspicion is not high but the ultrasound findings are equivocal; imaging must not delay surgical review where clinical suspicion is high (15).
- Suspected obstructive or complex Müllerian anomaly (29, 38, 72).
- Adnexal lesion with concerning features, for which the threshold for cross-sectional imaging is lower in prepubertal children: MRI has an important role for larger ovarian masses in this group (22), reflecting the higher risk of malignancy in adnexal lesions at this age (1).
- A pelvic cyst of uncertain origin, to distinguish an ovarian lesion from another pelvic or lower abdominal cyst such as a duplication cyst (6).
- Suspected deep endometriosis where transvaginal or transrectal ultrasound is not appropriate or is declined: MRI has identified endometriosis in adolescents whose earlier ultrasound raised no suspicion, although deep lesions are over-called on MRI even by experienced radiologists (76).

Three-dimensional transabdominal ultrasound may reduce the need for MRI, and in one pediatric series confirmatory MRI after a diagnostic 3D study added no anatomical information (19). European consensus calls three-dimensional ultrasound the reference standard for genital anomalies, with the transabdominal or transrectal route specified where the transvaginal approach is not available (20). The same guidance and the pediatric literature treat MRI as first-line in adolescents (20, 38, 72). It therefore remains unclear when three-dimensional ultrasound alone is sufficient.

### 4.6 Renal imaging in suspected Müllerian anomalies

The association between Müllerian and renal tract anomalies means renal imaging is warranted whenever a uterovaginal malformation is found or suspected (21, 38), and urinary tract investigation is considered mandatory (20). Its strength varies by anomaly, and renal imaging is strongly recommended for Müllerian agenesis and for unilateral obstructed vaginal and uterine anomalies: renal anomalies accompany Müllerian agenesis in up to 40% of cases and ipsilateral renal agenesis or anomaly in as many as 95% of obstructed hemivagina, but for other anomalies the data are too sparse to give an incidence (74). Conversely, girls with a solitary functioning kidney, whether from unilateral renal agenesis or a multicystic dysplastic kidney, should be screened for Müllerian anomalies between thelarche and menarche, before an obstruction becomes symptomatic (51, 77).

## 5 Discussion, limitations and gaps in the literature

Overall, the evidence describes a consistent pattern of pelvic development from birth through adolescence. This foundation is nonetheless constrained by important gaps that limit the applicability of published normative data and point to priorities for future research.

### 5.1 Heterogeneity and methodological limitations

The most pervasive limitation is the heterogeneity of published normative studies. Reported reference ranges vary with sample size, population demographics, imaging technology, measurement technique, and whether data are reported by chronological age, pubertal stage, or both. Prior normative data have themselves been described as outdated and limited by sample size (12)**.** Measurement method is itself a source of variability. Uterine length is not measured consistently: some studies report the corpus alone, others include the cervix (40). Ovarian volume is modality-dependent: ellipsoid and 3D estimates agree closely, but MRI yields systematically smaller volumes (11). Reported volumes also depend on the ellipsoid constant: the simplified formula (× 0.5) (41, 42) and the geometric prolate-ellipsoid formula (× π/6) (8, 14) differ by roughly 5% for identical diameters, so thresholds should be read with the source formula in mind. Cohorts differ, too, in whether they exclude polycystic-appearing ovaries from their centiles, as Bridges does, a study that also reported an interobserver coefficient of variation of about 11% (10). Few normative studies quantify reproducibility at all, and those that do differ widely: under 5% in one series (61), 15% in another (39), and 0 to 32% for linear measurements, with larger errors at larger dimensions (9). Pooled normative models are an important advance (13, 58), but inherit the heterogeneity of their source data.

### 5.2 Reliance on older studies and limited longitudinal evidence

Many foundational normative studies date from the 1980s and 1990s (10, 42, 45, 46, 56, 57); conducted on now-superseded technology, their measurements may not be comparable to modern high-resolution imaging (43). Endometrial detectability before puberty is itself equipment-dependent: older, lower-frequency studies frequently detected no echo (35, 47), whereas modern studies see a thin midline echo in essentially all prepubertal girls, though usually too thin to measure (14, 55). Longitudinal studies following the same girls from infancy through adolescence remain uncommon: even where imaging is nested in a birth cohort, the pelvis has typically been scanned only once (8, 39, 40, 78). Large-scale, multicenter longitudinal studies are needed to establish individual developmental trajectories and the range of normal intra-individual variation. Contemporary re-derivation of these values on current equipment is itself a research priority: the pooled normative models now available incorporate the foundational studies rather than testing them independently (13, 58).

### 5.3 Underrepresented age groups and populations

Normative data are sparsest at the youngest ages (12), and the interval between approximately 1 and 5 years remains least studied; even the neonatal-to-infancy transition has only recently received focused attention (49). This matters clinically, as it spans the resolution of minipuberty, when unexpected findings may cause undue concern.

Most data derive from European and North American populations, with some from Southern Europe (55, 60), South Asia (61, 62), Latin America (54) and the Middle East (63, 64); sub-Saharan Africa and East Asia remain substantially underrepresented. Further work is needed to determine how much of the reported variation reflects true population differences and how much results from differences in equipment or measurement technique. Data in special populations—Turner syndrome (45, 79), developmental disability (80), functional hypothalamic amenorrhea (81)—come from single-center studies not designed as normative references.

### 5.4 Limited evidence for specific ultrasound modalities

The vast majority of published normative data derive from 2D transabdominal ultrasound. Transrectal ultrasound has been evaluated in few studies (27), the single study of adolescent experience being an entirely Caucasian cohort whose authors call for multicultural designs (28). Evidence supporting transperineal ultrasound in children is emerging but remains limited (29). Three-dimensional ultrasound and MRI have been applied to uterine and ovarian volumetry in research cohorts (11, 37), but in the one head-to-head study against 2D transabdominal measurement they proved no more accurate, and the authors concluded transabdominal ultrasound should remain the standard (40). Uterine artery Doppler has been explored as a marker of pubertal onset (32), but no cut-off is validated and its clinical utility remains unproven (35). Prospective studies in community-based populations are needed to establish whether uterine artery Doppler adds diagnostic value beyond gray-scale morphometry in the evaluation of pubertal disorders, and whether the measurement can be obtained reliably in the youngest girls, in whom it most often fails.

### 5.5 Standardized reporting

No consensus reporting framework currently exists for pediatric and adolescent pelvic ultrasound. The one domain with a formal template is imaging of suspected disorders of sex development, for which the European Society of Pediatric Radiology recommends a systematic examination, cautious and neutral wording, and deferral of any conclusion to the end of the study (30), a useful model for pediatric gynecological reporting more broadly. Structured reporting has otherwise been trialed only for adnexal-mass risk stratification: adult classification systems have been tested in pediatric cohorts, but for O-RADS early results conflict and no prospective pediatric-specific validation yet exists (82, 83), while IOTA simple rules and benign descriptors show promising but unvalidated retrospective performance (1, 21). This reliance on adult-derived criteria extends beyond adnexal masses: conditions such as adenomyosis and endometriosis, historically considered rare in adolescents (15), are increasingly detected on ultrasound but have been characterized only in small, single-center series from expert centers applying adult diagnostic frameworks: the Morphological Uterus Sonographic Assessment (MUSA) criteria for adenomyosis and the International Deep Endometriosis Analysis (IDEA) criteria for endometriosis (36, 76). The development of standardized minimum reporting elements, including which measurements to obtain, how to report pubertal stage, and how to document normal variants, would improve the consistency and clinical utility of pediatric pelvic ultrasound reports.

## 6 Conclusion

A consistent theme runs through this review: measurement thresholds and diagnostic criteria derived from adults perform poorly in the developing pelvis, and interpretation is better grounded in age- and pubertal-stage-specific expectations than in fixed cut-offs. No single sonographic parameter reliably distinguishes adjacent pubertal stages, and no validated cut-off exists for pubertal onset (32, 35); adult criteria for polycystic ovarian morphology classify a third to nearly half of healthy post-menarcheal girls as abnormal (39, 70), which is why ultrasound is not recommended for diagnosing PMOS until eight years after menarche (68); the condition was separately renamed in 2026 (67); adult adnexal-mass criteria over-call physiological cysts in children (83).

Ultrasound remains first-line, and its value depends on the interpreter's familiarity with the developmental changes from the neonatal period through adolescence (1, 2). Interpretation is, for the most part, straightforward: assess the child in front of you against her pubertal stage and hormonal context, and ask whether the appearance is expected for that stage, recognizing the wide but normal spectrum of development at every age. This approach helps avoid unnecessary investigation of normal findings while ensuring that genuine abnormalities are recognized. Transabdominal ultrasound remains the mainstay, transvaginal, transrectal, transperineal, Doppler, and three-dimensional ultrasound each provide additional information in selected situations, while MRI is most useful when findings remain unclear or a complex anomaly is suspected. Future research should prioritize, first, prospective, multicenter, longitudinal studies in diverse populations, using standardized measurements and stage-based reporting, which would provide more contemporary reference data; and second, a standardized reporting framework for this population, which requires consensus rather than new cohorts and would make future studies comparable. Thereafter, studies should clarify the role of newer approaches such as three-dimensional ultrasound. In addition to further understanding of normative findings, similar studies should be designed, with particular focus on improving diagnosis of gynecological pathology in the pediatric and adolescent population.
