# Supplementary material for: Ultrasound modalities and age-specific normative findings in pediatric and adolescent gynecology
Source: Front Reprod Health. 2026 Oct 6;8:1953961. doi: 10.3389/frph.2026.1953961 (PMC13682351; doi:10.3389/frph.2026.1953961)
Supplement: Supplementary file 1 [file Table1.docx]

**Supplementary Table 1. PubMed search strings used in the literature search.**

| **No.** | **Theme** | **Search string** |
| --- | --- | --- |
| 1 | Reviews and overview | (("Ultrasonography"[Mesh] OR ultrasound[Title/Abstract] OR ultrasonograph*[Title/Abstract] OR sonograph*[Title/Abstract]) AND (pediatric[Title/Abstract] OR paediatric[Title/Abstract] OR child*[Title/Abstract] OR adolescen*[Title/Abstract]) AND (gynecol*[Title/Abstract] OR gynaecol*[Title/Abstract] OR pelvic[Title/Abstract])) AND review[Publication Type] NOT (pregnancy[Title/Abstract] OR obstetric*[Title/Abstract] OR infertility[Title/Abstract]) |
| 2 | Normative findings and reference values | (("Ultrasonography"[Mesh] OR ultrasound[Title/Abstract] OR ultrasonograph*[Title/Abstract] OR sonograph*[Title/Abstract]) AND (pediatric[Title/Abstract] OR paediatric[Title/Abstract] OR child*[Title/Abstract] OR adolescen*[Title/Abstract] OR neonat*[Title/Abstract] OR infant*[Title/Abstract] OR prepubert*[Title/Abstract] OR pubert*[Title/Abstract]) AND (normative[Title/Abstract] OR "reference value"[Title/Abstract] OR "reference values"[Title/Abstract] OR "reference range"[Title/Abstract] OR "reference ranges"[Title/Abstract] OR nomogram*[Title/Abstract] OR "cervix-to-body ratio"[Title/Abstract] OR "fundal-cervical ratio"[Title/Abstract] OR measurement*[Title/Abstract] OR dimension*[Title/Abstract] OR volume[Title/Abstract] OR morpholog*[Title/Abstract]) AND (uterus[Title/Abstract] OR uterine[Title/Abstract] OR ovary[Title/Abstract] OR ovarian[Title/Abstract] OR cervix[Title/Abstract] OR endometri*[Title/Abstract] OR pelvic[Title/Abstract])) NOT (pregnancy[Title/Abstract] OR obstetric*[Title/Abstract] OR infertility[Title/Abstract]) |
| 3 | Development and puberty | (("Ultrasonography"[Mesh] OR ultrasound[Title/Abstract] OR ultrasonograph*[Title/Abstract] OR sonograph*[Title/Abstract]) AND ("mini-puberty"[Title/Abstract] OR minipuberty[Title/Abstract] OR pubertal[Title/Abstract] OR "pubertal development"[Title/Abstract] OR Tanner[Title/Abstract] OR menarche[Title/Abstract] OR "precocious puberty"[Title/Abstract]) AND (uterus[Title/Abstract] OR uterine[Title/Abstract] OR ovary[Title/Abstract] OR ovarian[Title/Abstract] OR cervix[Title/Abstract] OR endometri*[Title/Abstract] OR pelvic[Title/Abstract]) AND (pediatric[Title/Abstract] OR paediatric[Title/Abstract] OR child*[Title/Abstract] OR adolescen*[Title/Abstract] OR girl*[Title/Abstract])) NOT (pregnancy[Title/Abstract] OR obstetric*[Title/Abstract] OR infertility[Title/Abstract]) |
| 4 | Specific ultrasound modalities | ((transperineal[Title/Abstract] OR translabial[Title/Abstract] OR transrectal[Title/Abstract] OR Doppler[Title/Abstract] OR "color Doppler"[Title/Abstract] OR "colour Doppler"[Title/Abstract] OR "3D ultrasound"[Title/Abstract] OR "three-dimensional ultrasound"[Title/Abstract]) AND (pediatric[Title/Abstract] OR paediatric[Title/Abstract] OR child*[Title/Abstract] OR adolescen*[Title/Abstract] OR girl*[Title/Abstract]) AND (gynecol*[Title/Abstract] OR gynaecol*[Title/Abstract] OR pelvic[Title/Abstract] OR uterus[Title/Abstract] OR ovary[Title/Abstract] OR ovarian[Title/Abstract] OR Mullerian[Title/Abstract] OR Müllerian[Title/Abstract])) |
| 5 | Technique and patient preparation | ((technique*[Title/Abstract] OR protocol*[Title/Abstract] OR "image acquisition"[Title/Abstract] OR "bladder filling"[Title/Abstract] OR "patient preparation"[Title/Abstract] OR positioning[Title/Abstract] OR "scanning technique"[Title/Abstract]) AND (ultrasound[Title/Abstract] OR ultrasonograph*[Title/Abstract] OR sonograph*[Title/Abstract]) AND (pediatric[Title/Abstract] OR paediatric[Title/Abstract] OR child*[Title/Abstract] OR adolescen*[Title/Abstract]) AND (gynecol*[Title/Abstract] OR gynaecol*[Title/Abstract] OR pelvic[Title/Abstract] OR genital[Title/Abstract])) |
| 6 | Müllerian, urogenital and associated renal anomalies; adjunct MRI | (("Ultrasonography"[Mesh] OR ultrasound[Title/Abstract] OR sonograph*[Title/Abstract] OR MRI[Title/Abstract] OR "magnetic resonance"[Title/Abstract]) AND (pediatric[Title/Abstract] OR paediatric[Title/Abstract] OR child*[Title/Abstract] OR adolescen*[Title/Abstract]) AND (Mullerian[Title/Abstract] OR Müllerian[Title/Abstract] OR "uterine anomaly"[Title/Abstract] OR "uterine anomalies"[Title/Abstract] OR "uterine malformation"[Title/Abstract] OR "uterine malformations"[Title/Abstract] OR "genital tract anomaly"[Title/Abstract] OR "genital tract anomalies"[Title/Abstract] OR "genital tract malformation"[Title/Abstract] OR "genital tract malformations"[Title/Abstract] OR "urogenital anomaly"[Title/Abstract] OR "urogenital anomalies"[Title/Abstract] OR "urogenital malformation"[Title/Abstract] OR "urogenital malformations"[Title/Abstract] OR "renal tract anomaly"[Title/Abstract] OR "renal tract anomalies"[Title/Abstract] OR "associated renal anomaly"[Title/Abstract] OR "associated renal anomalies"[Title/Abstract])) |
| 7 | Ovarian normal variants | (("physiologic ovarian cyst"[Title/Abstract] OR "physiologic ovarian cysts"[Title/Abstract] OR "physiological ovarian cyst"[Title/Abstract] OR "physiological ovarian cysts"[Title/Abstract] OR "functional ovarian cyst"[Title/Abstract] OR "functional ovarian cysts"[Title/Abstract] OR "follicular cyst"[Title/Abstract] OR "follicular cysts"[Title/Abstract] OR "asymmetric ovary"[Title/Abstract] OR "asymmetric ovaries"[Title/Abstract]) AND (ultrasound[Title/Abstract] OR ultrasonograph*[Title/Abstract] OR sonograph*[Title/Abstract]) AND (pediatric[Title/Abstract] OR paediatric[Title/Abstract] OR child*[Title/Abstract] OR adolescen*[Title/Abstract] OR girl*[Title/Abstract])) |
| 8 | Cervical and general normal variants and pitfalls | (("nabothian cyst"[Title/Abstract] OR "nabothian cysts"[Title/Abstract] OR "normal variant"[Title/Abstract] OR "normal variants"[Title/Abstract] OR pitfall*[Title/Abstract] OR overdiagnos*[Title/Abstract]) AND (ultrasound[Title/Abstract] OR ultrasonograph*[Title/Abstract] OR sonograph*[Title/Abstract]) AND (pediatric[Title/Abstract] OR paediatric[Title/Abstract] OR child*[Title/Abstract] OR adolescen*[Title/Abstract] OR girl*[Title/Abstract]) AND (gynecol*[Title/Abstract] OR gynaecol*[Title/Abstract] OR cervix[Title/Abstract] OR uterus[Title/Abstract] OR ovarian[Title/Abstract] OR ovary[Title/Abstract])) |

*Searches were run in PubMed between June and July 2026 and are reproduced here as run. Filters for English language and humans were applied through the PubMed interface and are not embedded in the strings shown; running a string without these filters returns a larger set. No publication date limits were applied. Exclusion of pregnancy, obstetric and infertility literature was applied at the search stage in searches 1, 2 and 3 only. MeSH, Medical Subject Headings; MRI, magnetic resonance imaging; 3D, three-dimensional.*
